# A Comparative Study of the ReCell^®^ Device and Autologous Split-Thickness Meshed Skin Graft in the Treatment of Acute Burn Injuries

**DOI:** 10.1093/jbcr/iry029

**Published:** 2018-05-24

**Authors:** James Hill Holmes IV, Joseph A Molnar, Jeffrey E Carter, James Hwang, Bruce A Cairns, Booker T King, David J Smith, C Wayne Cruse, Kevin N Foster, Michael D Peck, Rajiv Sood, Michael J Feldman, Marion H Jordan, David W Mozingo, David G Greenhalgh, Tina L Palmieri, John A Griswold, Sharmila Dissanaike, William L Hickerson

**Affiliations:** 1 Wake Forest Baptist Medical Center, Winston-Salem, North Carolina; 2 University Medical Center, New Orleans, Louisiana; 3 University of Alabama-Birmingham, Birmingham, Alabama; 4 University of North Carolina, Chapel Hill, North Carolina; 5 U.S. Army Institute for Surgical Research, Fort Sam Houston, Texas; 6 University of South Florida, Tampa, Florida; 7 Maricopa Health System, Phoenix, Arizona; 8 University of Indiana, Indianapolis, Indiana; 9 Virginia Commonwealth University, Richmond, Virginia Commonwealth University, Richmond, Virginia; 10 MedStar Washington Hospital Center, Washington, District of Columbia; 11 University of Florida, Gainesville, Florida; 12 University of California Davis, Sacramento, California; 13 Texas Tech University Health Sciences Center, Lubbock, Texas; 14 University of Tennessee, Memphis, Tennessee

## Abstract

Early excision and autografting are standard care for deeper burns. However, donor sites are a source of significant morbidity. To address this, the ReCell^®^ Autologous Cell Harvesting Device (ReCell) was designed for use at the point-of-care to prepare a noncultured, autologous skin cell suspension (ASCS) capable of epidermal regeneration using minimal donor skin. A prospective study was conducted to evaluate the clinical performance of ReCell vs meshed split-thickness skin grafts (STSG, Control) for the treatment of deep partial-thickness burns. Effectiveness measures were assessed to 1 year for both ASCS and Control treatment sites and donor sites, including the incidence of healing, scarring, and pain. At 4 weeks, 98% of the ASCS-treated sites were healed compared with 100% of the Controls. Pain and assessments of scarring at the treatment sites were reported to be similar between groups. Significant differences were observed between ReCell and Control donor sites. The mean ReCell donor area was approximately 40 times smaller than that of the Control (*P* < .0001), and after 1 week, significantly more ReCell donor sites were healed than Controls (*P* = .04). Over the first 16 weeks, patients reported significantly less pain at the ReCell donor sites compared with Controls (*P* ≤ .05 at each time point). Long-term patients reported higher satisfaction with ReCell donor site outcomes compared with the Controls. This study provides evidence that the treatment of deep partial-thickness burns with ASCS results in comparable healing, with significantly reduced donor site size and pain and improved appearance relative to STSG.

Each year, nearly 500,000 Americans suffer from acute thermal burn injuries requiring medical treatment, resulting in approximately 40,000 hospitalizations and 3400 deaths.^1–3^ Burn treatment is dictated by the depth and extent of the injury, with deeper and more extensive injuries requiring early excision and timely treatment with autologous split-thickness skin grafts (STSG) to achieve definitive closure and optimize clinical outcomes. Although the use of STSG is considered standard treatment, grafting is associated with significant pain, pruritus, infection, dyschromia, dyspigmentation, delayed healing, and hypertrophic scarring.^4,5^ Furthermore, in large TBSA injuries, donor site availability is a limitation for rapid wound closure using traditional skin grafting techniques.

The clinical benefits of earlier intervention for burn wounds are well recognized and include increased survival, reduced hospital length of stay, decreased hypertrophic scarring, decreased pain duration, and reduced infection-related complications.^6–11^ The realization that a treatment in which minimal split-thickness donor skin could be used to achieve definitive closure for burn wounds in a clinically advantageous time frame led to the investigation of strategies to harness the healing potential of the patient’s own skin.

The ReCell^®^ Autologous Skin Cell Harvesting Device (ReCell, Avita Medical, Valencia, CA, USA) was designed for point-of-care processing of a small split-thickness skin sample to produce an autologous skin cell suspension (ASCS). Using minimal donor skin with an effective treatment area expansion ratio of up to 80:1, the ASCS induces rapid epidermal regeneration and re-epithelialization. The ReCell device consists of a stand-alone, battery operated unit, a proprietary enzyme solution, buffer solution, sterile surgical instruments, and spray applicators to be used at the point-of-care, with no culturing processes involved in the procedure.

Preclinical data demonstrate that ASCS contains a mixed population of skin cells found at the dermoepidermal junction of predominantly keratinocyte, fibroblast, and melanocyte phenotypes.^12^ ReCell-generated ASCS has been used to treat a wide variety of wound conditions such as burns,^13,14^ STSG donor sites,^15^ chronic wounds,^16,17^ hypopigmented scars,^18^ vitiligo,^19,20^ and large congenital melanotic nevus.^21^ A randomized study conducted by Gravante et al demonstrated that deep partial-thickness burns treated with ASCS produced using the ReCell device showed similar results to standard skin grafting, with the use of significantly less donor skin. Additionally, subjects treated with ASCS reported experiencing significantly less pain than patients treated with meshed STSG.^13^

In burn wounds, the ReCell device is intended to generate ASCS that retains the known performance attributes of a STSG, while minimizing donor site morbidity. The aims of this study were to demonstrate similar definitive wound closure results using ASCS when compared with STSG and demonstrate improved donor site healing with the ReCell device.

## MATERIALS AND METHODS

### Study Design

This was a multicenter, prospective, within-patient controlled, randomized clinical trial conducted under a U.S. Food and Drug Administration (FDA) Investigational Device Exemption (NCT01138917). For each patient, two similar areas within a burn injury were treated according to random assignment, resulting in a control wound treated with a 2:1 meshed STSG and a ReCell wound treated with ASCS. Donor skin for each treatment was harvested from separate but similar uninjured areas. All study wounds were photographically documented throughout the 52-week trial. Before study initiation, the protocol was approved by the U.S. FDA, the U.S. Army Medical Research and Materiel Command Human Research Protection Office (USAMRMC HRPO), and Institutional Review Boards (IRBs) at each individual site.

### Patient Selection

Patients ranging in age from 18 to 65 years were eligible for study enrollment if they presented with an acute, deep partial-thickness thermal burn from 1% to 20% TBSA that required autografting for definitive closure. A minimum burn injury treatment area of 200 to 640 cm^2^ was required, allowing for treatment of two separate or contiguous 100 to 320 cm^2^ areas. Exclusion criteria for the study included the following: burn injuries caused by chemicals, electricity, and/or radioactive substances; burn injuries <1% or >20% TBSA; treatment areas involving the head, face, neck, hands, feet, genitalia, or over joints; presence of pre-existing local or systemic infections; antibiotic treatment for >48 hours before autografting for other than prophylactic reasons; hypersensitivity to trypsin; a pre-existing chronic condition that in the opinion of the investigator might interfere with wound healing (eg, malignancy, diabetes, or autoimmune disease); inability to follow the protocol; medications that could interfere with wound healing (ie, corticosteroids); other concurrent conditions that in the opinion of the investigator might compromise patient safety or study objectives; and pregnant or breast-feeding women or women who wished to become pregnant during the length of study participation.

### Randomization

Within-subject allocation of treatments to selected burn wounds was performed at random, using a predetermined random assignment of treatments. Following excision and confirmation of deep partial-thickness depth, and before randomization, the surgeon marked each area as site “A” or site “B.” Wound marking was performed using a sterile marker, allowing easy identification of the proposed treatment sites. An envelope was opened indicating treatment allocation.

### Donor Skin Harvesting

For the Control wound donor site, an autograft was harvested from a preselected, noninjured skin site according to standard of care (SOC), typically at a thickness of 0.008 to 0.010” but no less than 0.006”. For the ReCell wound, a thin split-thickness skin sample was harvested at a second donor site discrete from the Control wound donor site such that the two wounds could be dressed and assessed in isolation. The ReCell wound donor site was harvested at a depth of 0.006 to 0.008” with the average size of 4.7 cm^2^. Before processing donor skin with the ReCell device, the skin was trimmed down to no greater than 4 cm^2^ (average 3.9 cm^2^) so that it would cover up to 320 cm^2^ or up to 1:80 expansion ratio.

### Burn Site Preparation and Treatment

The treatment sites were excised to remove all nonviable tissue, and hemostasis was achieved per SOC. The surgeon verified that the wound was deep partial-thickness and contained viable dermis throughout the wound base.

### ASCS Treatment

Treatment using the ReCell device was performed in accordance with the manufacturer’s instructions. Briefly, the split-thickness skin sample was transferred to the warmed proprietary enzyme solution for a period of 15 to 20 minutes to promote breakdown of cell-cell and cell-matrix adhesions, including those at the dermoepidermal junction. The skin sample was then removed from the enzyme, placed on the sterile tray of the device, and tested to determine whether the epidermis could be freely separated from the dermal tissue. When the tissue layers could be freely separated, the skin was rinsed in buffer solution and returned to the sterile tray of the device, dermal side down. Both the dermal and epidermal layers were vigorously scraped with a scalpel blade to completely disaggregate the skin sample. Following complete disaggregation, the cells were suspended in a buffer solution, filtered, drawn into the provided application syringe, and applied over the area randomized to receive ASCS treatment.

### Meshed STSG Treatment

The harvested STSG was meshed using a skin meshing system of choice at a ratio of 2:1. The prepared 2:1 meshed STSG was maintained in saline moistened gauze until placement on the prepared wound bed. The autograft was secured in place using sutures or staples at the surgeon’s discretion.

### Postoperative Care

After applying the ASCS and the 2:1 meshed autograft, both treatment sites were covered with a nonadherent, low-absorbent, small pore dressing (Telfa^™^ Clear Wound Dressing, Covidien, Minneapolis, MN). A secondary dressing of Xeroform^™^ Petrolatum Gauze Dressing (Covidien, Minneapolis, MN) was placed over the Telfa Clear primary dressing. Additional padding of gauze and a crepe bandage were used at the surgeon’s discretion for exudate and protection. The Telfa Clear primary dressing remained in place for a minimum of 6 to 8 days and was not manipulated until the first postoperative study visit unless medically necessary. Secondary dressings were replaced as needed. Telfa Clear was also used as the primary dressing for donor sites, with secondary dressing selection at the discretion of the surgeon. Most frequently, Xeroform was used as a secondary dressing for donor sites.

### Study Endpoints

Primary effectiveness endpoints were 1) the incidence of wound closure (≥95% re-epithelialization) of the treated sites at 4 weeks and 2) the incidence of complete donor site healing at 1 week (100% re-epithelialization), as determined by the surgeon. Percent re-epithelialization of the treatment sites was assessed using standardized planimetry/tracing procedures. The tracings were uploaded to a central reading facility (Canfield Scientific, Parsippany, NJ) for calculation of percent re-epithelialization.

Secondary endpoints included pain, visual appearance, and scarring. Pain and visual appearance were assessed using visual analog scales (VAS-style ratings, 0–100). For both the treatment and donor sites, pain was assessed at weeks 1, 2, 3, 4, 8, and 16, and visual appearance was assessed at weeks 16, 24, and 52. Investigators used the Vancouver Scar Scale (VSS) to evaluate scarring at weeks 16, 24, and 52 for both the treatment and donor sites.

### Safety Analyses

All adverse events were summarized and tabulated by treatment, system organ class, and preferred term. Adverse events were also evaluated by severity and if they were device-related adverse events. Additionally, descriptive statistics were provided for the following safety variables including infection at recipient sites, infection at donor sites, and graft loss at recipient site.

### Statistical Methods

The study was designed to investigate the clinical performance of ASCS relative to Control STSG, for the treatment of deep partial-thickness burn injuries. Coprimary effectiveness objectives were to test noninferiority of the incidence of ASCS-treated site wound closure at 4 weeks when compared with that of the Control, and the superiority of the incidence of ReCell donor site healing at 1 week when compared with that of the Control.

For the null hypothesis to be rejected and noninferiority to be established, the lower limit of the observed one-sided 97.5% confidence interval was expected to exceed −0.100 (a prespecified 10% noninferiority margin) with 81% power when the expected difference (*π*_T_ − *π*_S_, Δ_1_) is 0.000, the proportion discordant (η = *π*_10_ – *π*_01_) is 0.100, and the proportion of both yes (*π*_11_) is 0.750 for a population of 90 subjects. Results are based on 1000 simulations using the Newcombe–Wilson score method to construct the confidence interval.^22^

The noninferiority margin of 10% was determined based on input from experienced burn surgeons and considered two factors: 1) it is normal for there to be minor variation in the rate of healing even for the same patient and 2) the clinical benefit that is realized at the donor site (ie, a smaller, faster-healing donor site) provides balance for acceptance of a 10% noninferiority margin for recipient site healing.

In the comparison of the coprimary endpoint of superiority of donor site wound healing between the ReCell donor site and the STSG donor site, it was assumed that at least 95% of the ReCell donor sites would be healed at 1 week, whereas 40% of the STSG donor sites would remain unhealed. For these assumptions, a sample size of 42 pairs will have 95% power to detect a difference in proportions of 0.350 when the proportion of discordant pairs is expected to be 0.500 and the method of analysis is a McNemar’s test of equality of paired proportions with a 0.050 two-sided significance level.

Thus, a recruitment target was established aiming to yield 90 evaluable subjects such that the noninferiority analysis is sufficiently powered.

### Populations for Analysis

**Figure 1.**
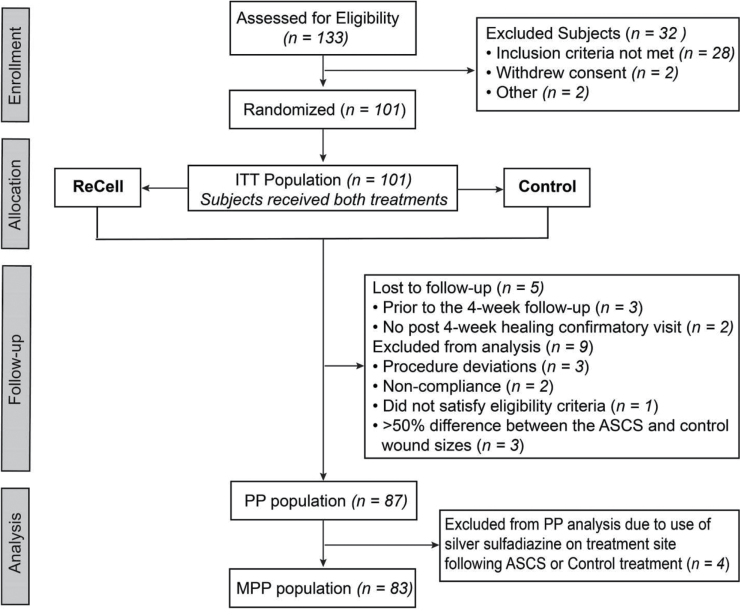
One hundred one consenting subjects meeting inclusion and exclusion criteria were enrolled into the study. Eligible burn injury sites were randomized to receive ReCell or Control (2:1 meshed skin graft) treatment and followed over a 52-week period (intention to treat [ITT] population). Of these subjects, 87 were evaluated within the per protocol (PP) population analysis and 83 were part of the modified per protocol (MPP) population analysis.

Study populations (Figure 1) were defined as follows: 1) Intent-to-treat population (ITT): all randomized subjects (n = 101) which was used for demographics, evaluation of safety, superiority of donor site healing, and scar ratings; 2) Per-protocol (PP) population(n = 87): ITT subjects who receive both study treatments in accordance with the randomization, completed 4 weeks of follow-up and had no major protocol deviations up to 16 weeks which was used for evaluation of patient self-report of pain and appearance (of both treatment and donor areas); and 3) Modified Per-protocol (MPP) population (n = 83): PP subjects who did not undergo concomitant therapy known to impair wound healing (ie, use of topical silver sulfadiazine [SSD]). The ITT and PP populations were prospectively defined. The MPP population was defined post hoc to resolve concern that the PP population included subjects with results confounded by the use of a cytotoxic agent concomitant with the ASCS. At the initiation of the study, SSD use was not contraindicated, as its use was not anticipated; therefore, it was determined that excluding these patients from the PP population was not appropriate within the context of an FDA clinical trial.

The statistical analysis of the data obtained from the study was conducted by an independent third-party (BioStat International, Tampa, FL) using SAS Version 9.3. Continuous variables were summarized using descriptive statistics, specifically the mean, median, standard deviation, minimum, and maximum. Categorical variables were summarized by frequencies and percentages.

## RESULTS

Between May 2010 and August 2014, 133 patients were assessed for eligibility across 12 burn centers within the United States, and 101 patients were enrolled in the study. The mean patient age was 39.5 ± 13.1 years, and 84% of the patients were male, whereas the majority of burns (77%) were due to fire/flame-related injuries (Table 1). Subjects received treatment, on average, 7.1 ± 3.0 days following their burn injury, allowing time for confirmation that the depth of injury was such that autografting was medically indicated (Table 1).

**Table 1.** Subject demographics and burn injury characteristics

Age (y)	39.5 ± 13.1 (Mean ± Stdev)
	18.2–63.5 (Range)
Sex	84.2% Male
	15.8 % Female
Race/Ethnicity	58.4% White
	19.8% Black
	18.8% Hispanic
Etiology	77.2% Fire/flames
	14.9% Hot water/steam
	6.9% Excessive heat
	1.0% Fire/flames/excessive heat
Treatment postburn injury (d)	7.1 ± 3.0 (Mean ± Stdev)
	2–19 (Range)
TBSA (%)	10.0 ± 4.5 (Mean ± Stdev)
	3–20 (Range)

Of the 101 patients enrolled (ITT), 87 subjects completed the 52-week follow-up without major protocol deviations (PP). The analysis population for incidence of definitive closure of the treatment sites (MPP) consisted of 83 subjects, with four subjects being excluded from the PP population who had SSD applied at the ASCS-treated sites following ASCS application (Figure 1).

**Figure 2.**
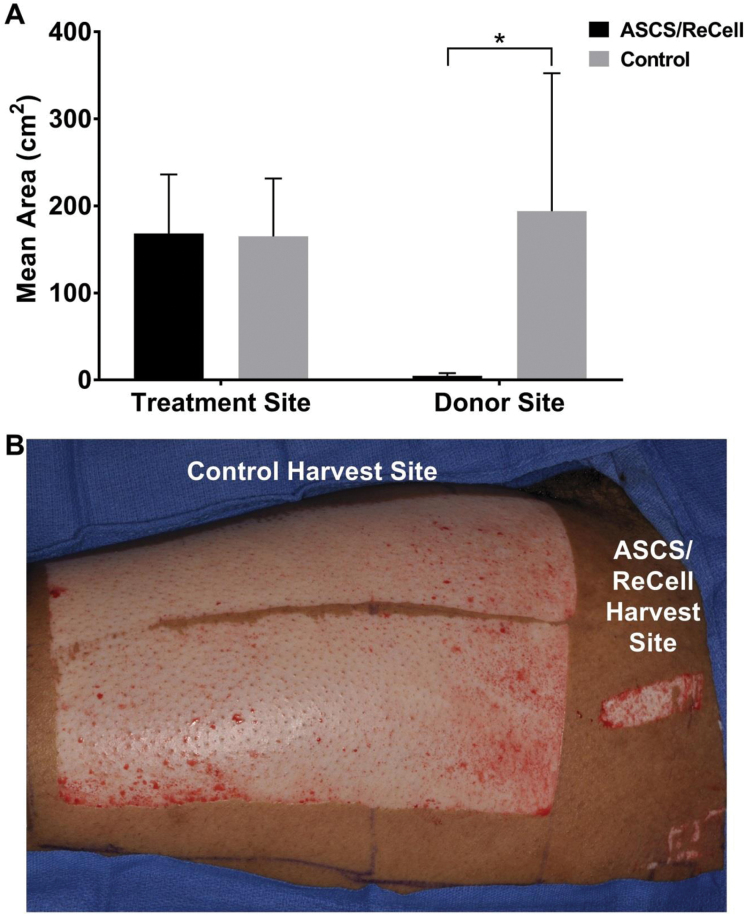
Autograft Sparing Analysis. (A) Treatment and donor site wound areas. The mean size of treatment sites was 168.2 ± 68.0 cm^2^ (ASCS/ReCell) and 165.0 ± 66.5 cm^2^ (Control). Mean size of donor sites was 4.7 ± 3.2 cm^2^ (ASCS/ReCell) and 194.1 ± 158.5 cm^2^ (Control). * indicates a statistical difference with *P* < .0001. (B) Clinical case example of donor site harvest areas for ASCS/ReCell skin sample and Control split-thickness skin graft.

The two treatment areas were comparable with respect to anatomic location and size, with the ASCS treatment area averaging 168 cm^2^ and control treatment area 165 cm^2^ (Figure 2). The average area of the donor sites for ASCS was approximately 40 times smaller than the average area of the donor sites for the control treatment (4.7 ± 3.2 cm^2^ vs 194.1 ± 158.5 cm^2^, respectively; *P* < .0001; Figure 2), translating to a donor site size reduction of 97.5%. The average amount of donor tissue required to prepare ASCS was 3.9 ± 0.3 cm^2^ as it was necessary to trim the donor tissue to obtain a final size of up to 4 cm^2^ (to cover an area up to 320 cm^2^).

### Treatment Sites

**Figure 3.**
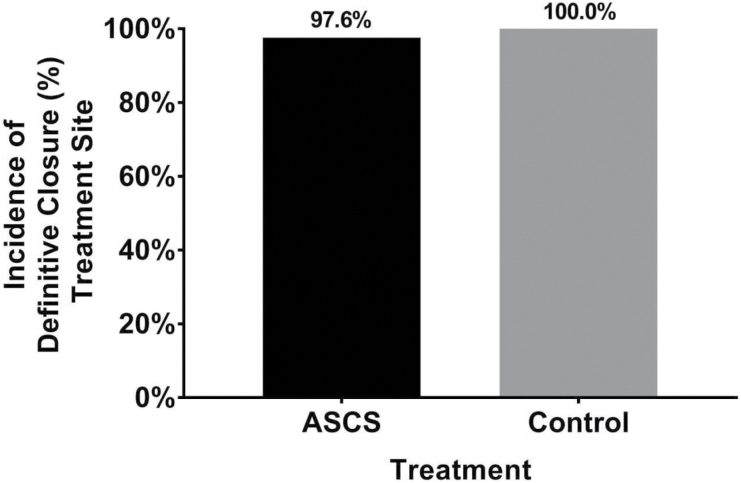
Incidence of definitive closure at treatment site. The primary effectiveness analyses to evaluate noninferiority of the incidence of treatment site wound closure with autologous skin cell suspension (ASCS) compared with Control treatment at week 4 was performed on the MPP population. The proportion of subjects with treatment site wound healing at week 4 was 97.6% for ASCS and 100% for Control with a −2.4% difference in proportions and a 95% CI: −8.4%, 2.3%. As the lower bound of the 95% CI is greater than the predefined −10% NI margin, noninferiority was established.

Both treatments were clinically effective in healing >97% of the treated burns by week 4. The incidence of definitive closure, as defined as ≥95% re-epithelialization, was 97.6% (81/83) for the ReCell-generated ASCS treated site and 100% (83/83) for the control STSG–treated site (Figure 3). The difference in proportions between the two groups was −2.4% (95% CI: −8.4% to 2.3%). Therefore, the healing of ASCS-treated sites was statistically comparable to those sites treated with a 2:1 meshed STSG, thus substantiating the noninferiority primary effectiveness endpoint.

**Figure 4.**
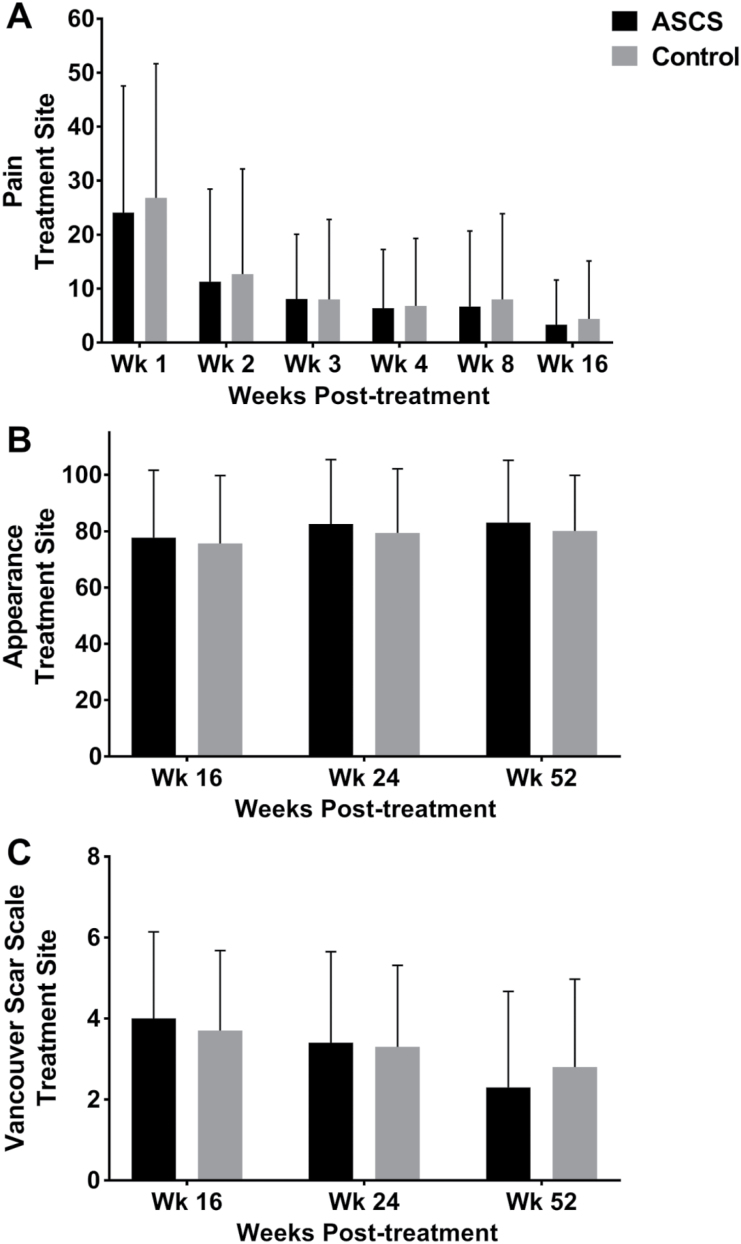
Pain and visual appearance assessments at treatment site. (A) Pain was assessed at weeks 1, 2, 3, 4, 8, and 16 by the VAS Pain Scale. No difference in pain was reported between the autologous skin cell suspension (ASCS) and Control treatment sites. Visual appearance of the treatment site (B) and scarring at the treatment site (C) was assessed at weeks 16, 24, and 52. (B) No difference in visual appearance (VAS Appearance Scale) was reported between the ASCS and Control treatment sites. (C) No difference in scarring (Vancouver Scar Scale) was reported between the ASCS and Control treatment sites.

Additionally, subject-reported pain at the treatment site during the first 16 weeks was not significantly different between the ASCS and control sites (Figure 4A). Similarly, long-term results at 16, 24, and 52 weeks showed no difference in subject satisfaction with appearance (Figure 4B) or in scarring (Figure 4C) at the ASCS-treated sites compared with the control sites. Following treatment site healing, no late-term wound breakdown was reported.

### Donor Sites

**Figure 5.**
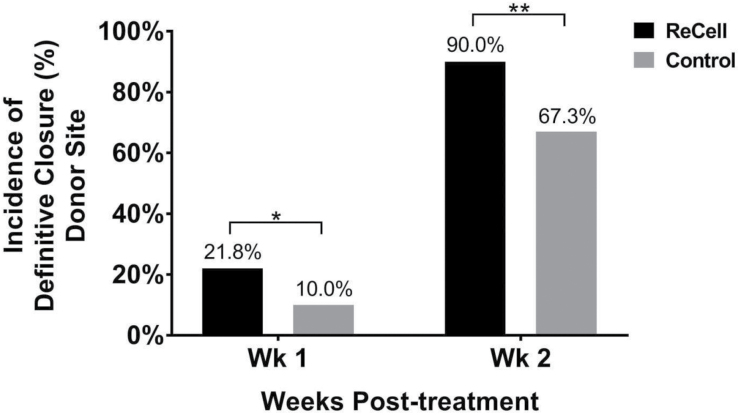
Incidence of definitive closure at donor site. Wound healing of the donor sites taken for the ReCell treatment and the Control treatment was assessed at the 1- and 2-week visits. Significantly more donor sites taken for ReCell were healed when compared with the Control donor site wounds at weeks 1 and 2 (Week 1: * indicates *P* < .05; Week 2: ** indicates *P* < .001).

**Figure 6.**
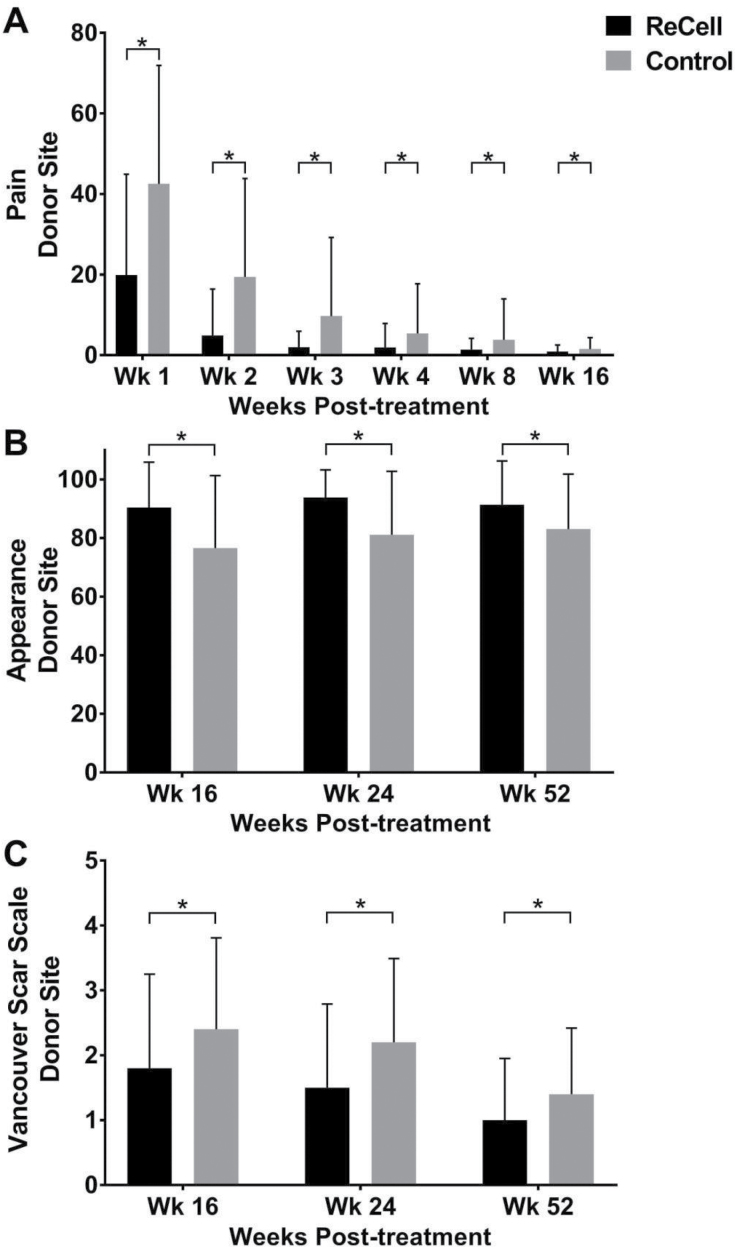
Pain and visual appearance assessments at donor site. (A) Pain was assessed at weeks 1, 2, 3, 4, 8, and 16 by the VAS Pain Scale. Subjects reported statistically significantly less pain at the ReCell donor site than the Control donor site through the 8-week visit (* indicates *P* ≤ .005 at each interval). Visual appearance of the donor site (B) and scarring at the donor site (C) was assessed at weeks 16, 24, and 52. Patients expressed significantly greater satisfaction with the visual appearance (VAS Appearance Scale) of the ReCell donor sites compared with the Control donor sites at the weeks 16, 24, and 52 (* indicates *P* ≤ .005 at each interval). Reduced scarring (Vancouver Scar Scale) was reported at the ReCell donor sites compared with the Control donor sites (* indicates *P* ≤ .005 at each interval).

At 1 week, the incidence of donor site healing in the ReCell group was shown to be superior to the control group (21.8% vs 10.0%; *P* = .04, Figure 5), thus validating the superiority primary effectiveness endpoint. At week 2, the incidence of donor site healing in the ReCell group was also statistically significant in favor of the ReCell donor site (90.0% vs 67.3%; *P* < .001) with an odds ratio of 4.3. Additionally, this superiority persisted and subjects reported a statistically significant, and clinically meaningful, reduction in pain at the ReCell donor site compared with the Control donor site through the week-8 visit (*P* ≤ .005 at each interval, Figure 6A). Long-term assessments at weeks 16, 24, and 52 showed that subjects expressed greater satisfaction with the visual appearance of the ReCell donor sites compared with the Control donor sites (*P* ≤ .005 at each interval, Figure 6B). Reduced scarring was also reported at the ReCell donor sites compared with the Control donor sites (*P* ≤ .005 for each time point, Figure 6C).

### Safety

All safety analyses were conducted on the ITT population. The majority of adverse events (AEs), including treatment and donor sites, were mild in nature (83.3% and 91.3% for ASCS-treated sites and Control-treated sites, respectively; Table 2), and there were no subject deaths reported (Table 2). The greater incidence of AEs noted at the ASCS-treated sites is primarily attributed to the application of SSD following ASCS application and reinjury at the recipient site due to lack of protective dressings/garments following initial re-epithelialization. There were no events of late wound breakdown at either the ASCS- or Control-treated sites. Overall, AEs reported for ASCS-treated sites were typical for the type of injury sustained by subjects with burn wounds requiring skin grafting procedures.

**Table 2.** Summary of adverse events

Adverse Events	ReCell n* (%)	Control n* (%)	ReCell vs Control *P*^†^
No Adverse Events	65 (64.4%)	78 (77.2%)	0.0044
Mild	30 (29.7%)	21 (20.8%)	1.0000
Moderate	5 (5.0%)	1 (1.0%)	
Severe	1 (1.0%)	1 (1.0%)	
Treatment Site Adverse Events			
Total (Any primary system organ class)	36 (35.6%)	22 (21.8%)	0.0013
Total Infections and Infestations	3 (3.0%)	2 (2.0%)	1.0000
Injury, Poisoning, and Procedural Complications	10 (9.9%)	2 (2.0%)	0.0215
Total Skin and Subcutaneous Tissue Disorders	26 (25.7%)	16 (15.8%)	0.0129
Device-Related Adverse Events			
Total (Any primary system organ class)	5 (5.0)	N.A.	
Skin graft failure	2 (2.0)	N.A.	—
Hypertrophic scar	3 (3.0)	N.A.	
Donor Site Adverse Events			
Total (Any primary system organ class)	3 (4.0)	6 (6.9)	0.2500
Total Infections and Infestations	0 (0.0)	1 (1.0)	0.5000
Injury, Poisoning, and Procedural Complications	0 (0.0)	1 (1.0)	—
Total Skin and Subcutaneous Tissue Disorders	3 (3.0)	4 (4.0)	0.5000

*Subjects with multiple occurrences of a preferred term is counted only once for that preferred term.

^†^ *P*-value obtained using McNemar’s test.

Five device-related AEs were reported as follows: two mild skin graft failures and three hypertrophic scarring (two mild and one moderate). However, for 2 of 3 subjects, hypertrophic scarring was reported at the Control site and other nonstudy wounds as well. Therefore, the relatedness of the scarring to the study device is questionable. Overall, two subjects underwent subsequent intervention for graft loss at the ReCell site (one regrafting procedure and one debridement with redressing). In both cases, the wounds were healed at 4 weeks and remained healed.

For the ReCell and Control donor sites, there were no differences in the incidence of AEs (4.0% vs 6.9%, respectively, *P* = .25). At the treatment sites, a greater number of total AEs occurred with the ReCell treatment than the Control treatment (35.6% vs 21.8%, respectively, *P* = .0013).

### Representative Case Example

**Figure 7.**
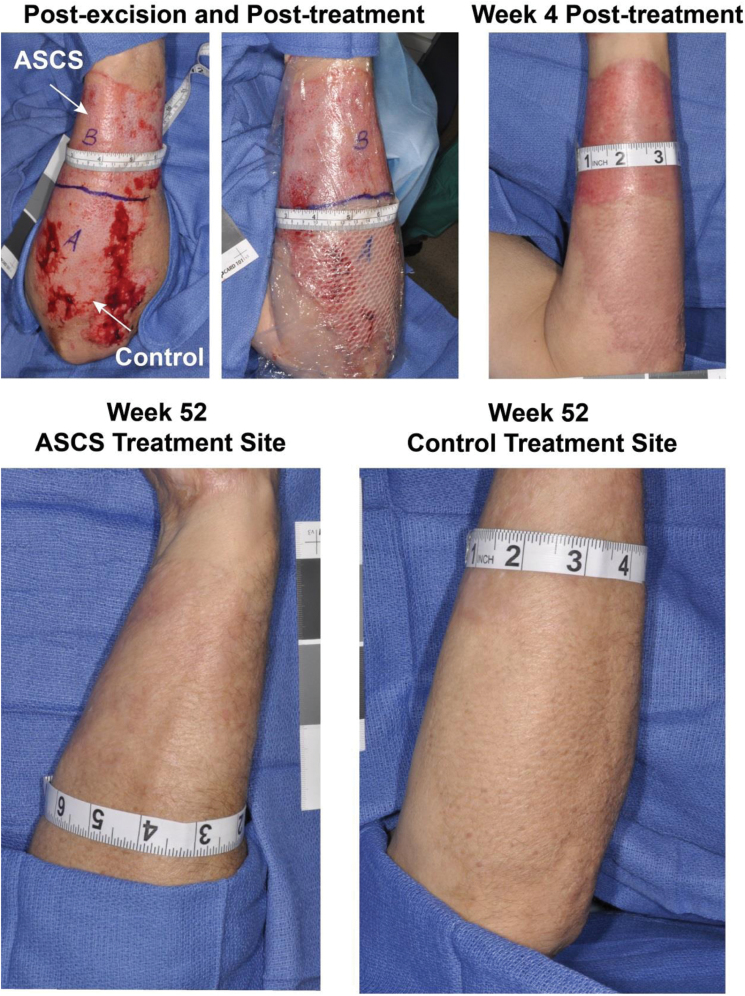
Clinical case. A 62-year-old white male sustained an 8.5% TBSA injury from fire/flames inclusive of an injury to the left forearm. The burn wound on the left forearm was excised and divided into two sections (A and B) and these sites were randomized as the autologous skin cell suspension (ASCS) or Control wounds. Site A received the control treatment (2:1 meshed STSG) and site B ASCS. Telfa Clear was used to cover both treated sites followed by Xeroform and bulky dressings. At 4 weeks, both treatment sites were healed. At 52 weeks, the color and pigment of the ASCS treatment site matched surrounding skin, whereas the Control treatment site was mildly mismatched in color and pigment.

A 62-year-old white male sustained an 8.5% TBSA flame burn inclusive of an injury to the left forearm (Figure 7). The burn was excised and divided into two sites (A and B), and these sites were randomized. The site assigned to ASCS was (B) and measured 185 cm^2^, whereas the site assigned to Control STSG was (A) and measured 121 cm^2^. A 90.25-cm^2^ STSG was harvested from the left anterior thigh at 0.010” thick, meshed 2:1, and applied to site A (control). A 4-cm^2^ skin sample was harvested from the medial thigh at 0.007” thick and processed using the ReCell device, with ASCS being applied to Site B. Telfa Clear was used to cover both of the treated sites and donor sites, followed by Xeroform and gauze dressings. One week following intervention, both the ASCS site and the control site were healed. At week 52, the color and pigment of the ASCS site matched surrounding skin, whereas the control site was mildly mismatched in color and pigment. The overall VSS for the control site was 2, whereas the ASCS-treated site was 0.

## DISCUSSION

In this study, it was demonstrated that the use of ASCS generated from the ReCell^®^ Autologous Cell Harvesting Device is a safe and effective alternative to conventional meshed autografting for the treatment of deep partial-thickness burns. It was demonstrated that, at 4 weeks, in a patient population receiving proper wound care, the incidence of healing was comparable between the treatment groups (97.6% for ASCS and 100% for Control), with no differences in pain, subject satisfaction with appearance, or scarring outcomes.

Additionally, ReCell donor sites demonstrated increased healing, decreased pain, improved subject satisfaction with appearance, and less scarring. It was demonstrated that the requirement for harvesting of donor skin may be 1/80th the size of the burn injury area treated when using the ReCell device, thus reducing the skin required for coverage by 97.5% relative to 2:1 meshed STSG. Furthermore, as shown in this study, the use of ReCell additionally decreases the donor site depth compared with standard grafting, likely contributing to the reduced pain and improved healing outcomes.

Using ReCell, the reduction of donor skin requirements gives rise to significant clinical benefits, offering surgeons a new strategy for the treatment of burn injuries. This is particularly true for patients having limited donor tissue availability, as well as for patients in whom the creation of larger donor sites may lead to significant morbidity. Additionally, for indeterminate depth injuries where skin grafting is intentionally delayed until it is certain that a graft is needed for definitive closure, the use of ReCell may be a solution for earlier intervention, as morbidity and complications associated with donor sites are reduced.

This study provides definitive evidence for the safe and effective use of ReCell in the treatment of deep partial-thickness burns. As part of the study design to mitigate the variability across groups, treatment was limited to an area of 320 cm^2^ in burns of 1% to 20% TBSA and excluded application to joints, hands, feet, genitalia, and faces. Although this study excluded these patients and treatment regions, previously published studies using ReCell demonstrate successful use in deep partial-thickness injuries for patients with varying TBSA and across various anatomic locations.^13,23,24^ In addition, ReCell treatment to the donor sites has previously been shown to significantly improve donor site healing.^15^ Therefore, based on the collective data, the authors conclude that the results demonstrated in this study may be extrapolated for the treatment of all deep-partial thickness thermal burns.

Further implications from this study include the importance of understanding the learning curve clinicians must overcome in using innovative cellular products. During this study, adverse events occurred that were not attributable to the ASCS application but were due to the failure of proper after care. As the cellular suspension contains disaggregated skin cells, it is critical that the environment is optimized to allow for the regeneration and maturation into a robust epidermal layer. Thus, following ASCS application, cytotoxic agents must be avoided and dressings and garments should be used to protect the newly formed skin.

## CONCLUSION

Overall, the study demonstrates that ASCS generated by the ReCell device is a viable alternative to STSG for the treatment of deep partial-thickness thermal burns. Definitive wound closure of ASCS-treated sites was comparable to sites treated with 2:1 meshed autograft. Patients benefitted from significantly improved incidences of donor site healing and reduced pain and morbidity of the associated smaller donor sites. Reducing the amount of donor skin required to achieve complete and definitive burn wound closure opens the possibility for treating extensive and complex burns sooner than the current SOC. These findings have potential implications for a paradigm shift in the approach used to achieve rapid and permanent closure of burn injuries. Furthermore, achieving definitive closure using less skin compared with standard autografting has the potential to decrease the number of surgical procedures required to achieve wound closure as well as reducing hospital length of stay, thus decreasing the overall costs related to the treatment of burn injuries.

## Funding

This study was sponsored by Avita Medical, the Biomedical Advanced Research and Development Authority (BARDA), and the Department of the Army, AFIRM 1 Contract #W81XWH-08-2-0032 (awarding and administering acquisition office: U.S. Army Medical Research Acquisition Activity, 820 Chandler Street, Fort Detrick MD 21702–5014). The study results and conclusions do not necessarily reflect the position or the policy of the Government, and no official endorsement should be inferred.
